# TransComb: genome-guided transcriptome assembly via combing junctions in splicing graphs

**DOI:** 10.1186/s13059-016-1074-1

**Published:** 2016-10-19

**Authors:** Juntao Liu, Ting Yu, Tao Jiang, Guojun Li

**Affiliations:** 1 School of Mathematics, Shandong University, Jinan, 250100 China; 2 Department of Computer Science and Engineering, University of California, Riverside, CA 92521 USA; 3 MOE Key Lab of Bioinformatics and Bioinformatics Division, TNLIST/Department of Computer Science and Technology, Tsinghua University, Beijing, China; 4 Institute of Integrative of Genome Biology, University of California, Riverside, CA 92521 USA

**Keywords:** RNA-seq, Transcriptome assembly, Alternative splicing, Splicing graph, Isoform

## Abstract

Transcriptome assemblers aim to reconstruct full-length transcripts from RNA-seq data. We present TransComb, a genome-guided assembler developed based on a junction graph, weighted by a bin-packing strategy and paired-end information. A newly designed extension method based on weighted junction graphs can accurately extract paths representing expressed transcripts, whether they have low or high expression levels. Tested on both simulated and real datasets, TransComb demonstrates significant improvements in both recall and precision over leading assemblers, including StringTie, Cufflinks, Bayesembler, and Traph. In addition, it runs much faster and requires less memory on average. TransComb is available at http://sourceforge.net/projects/transcriptomeassembly/files/.

**Electronic supplementary material:**

The online version of this article (doi:10.1186/s13059-016-1074-1) contains supplementary material, which is available to authorized users.

## Background

Recent research has revealed the immense complexity and diversity of transcriptomes in eukaryotes [1]. To understand the exact mechanism of alternative splicing in multi-exon protein coding genes, researchers have conducted both computational and experimental studies. A recent publication [2] showed that proteins translated along with alternative splicing can be captured by an electron microscope, which clearly provides some insights into the mechanism of alternative splicing. However, it remains a highly challenging task to elucidate the alternative splicing mechanism in eukaryotic species.

The technology of RNA-seq is rapidly changing our ability to explore the very complex transcriptomic landscape as it can provide unprecedented accuracy in quantifying mRNA expression levels [3]. This technology also accurately elucidates all splicing events, including those that are rare and expressed at a low level. This opens a new door to study the mechanism of complex diseases related to abnormal splicing or expression levels, such as cancers. However, it has been observed [4–6] that alternative splicing events may produce various isoforms in eukaryotic tissues and, moreover, there are many kinds of splicing events, including retained introns, skipped exons, and mutually exclusive exons, and some exons may even be partially spliced, so-called partial exons. In addition, an RNA-seq run may produce more than 200 million short reads, each of which consists of only 50–150 bp. Therefore, it is highly challenging to accurately assemble the huge amount of short reads into full-length transcripts computationally.

There are two main strategies for transcriptome assembly, genome-guided and de novo [3, 7]. Genome-guided assemblers, such as StringTie [8], Bayesember [9], Cufflinks [10], Scripture [11], IsoInfer [12], IsoLasso [13], iReckon [14], CEM [15], Traph [16], and CIDANE [17], usually first map short reads to a reference genome using an alignment tool, such as TopHat [18], TopHat2 [19], SpliceMap [20], or GSNAP [21], to cluster the reads into gene loci based on which the so-called splicing graph or overlap graph can be constructed for each individual cluster. Then, a well-studied mathematical model, e.g., minimum-cost minimum path cover, can be applied to search for transcript-representing paths in each splicing graph, as it does in Cufflinks [10]. As stated in [9], however, minimum-cost minimum path cover may not be a good choice because this strategy may lack a biological foundation and more complex solutions may better explain the full graph coverage. Pertea et al. [8] demonstrated that Scripture has very low precision because it tends to predict a large number of splice variants for each gene, most of which are false positives. StringTie uses a greedy algorithm to search for transcript-representing paths in splicing graphs without effectively solving the ambiguities caused by exons with multiple splicing junctions. Moreover, StringTie updates the current splicing graph based on estimated expression level of the current assembled transcript, which may lead to successively erroneous predictions once an erroneous assembled transcript is encountered because the splicing graph will be wrongly updated later on. Traph uses a minimum-cost flow model combined with a greedy algorithm in the assembling procedure, which is easily collapsed especially for complicated splicing graphs. Others, such as IsoLasso, IsoInfer, CEM and iReckon, predict transcripts and estimate their expression levels as well using regularization-based methods. However, these methods achieve sparsity by thresholding transcript expression levels, which results in penalizing low expression transcripts. Bayesembler is a very recently published probabilistic method for both transcriptome assembly and expression level estimation. It uses a probabilistic model to simulate the RNA sequencing process without penalizing lowly expressed transcripts. However, an enumeration strategy was employed in its procedure, which makes it extremely time consuming. CIDANE is a newly published assembler which was developed based on genome annotation. However, it is not included as a comparator in this study as TransComb and the other compared tools only take as input the alignment of RNA-seq data.

De novo assemblers, such as BinPacker [22], Bridger [23], Trinity [7], ABySS [24], SOAPdenovo-Trans [25], Oases [26], and IDBA-Tran [27], assemble transcripts directly from short reads without using a reference genome. However, the presence of gene homology, splicing event diversity, and sequence coverage unevenness makes the de novo strategy even more challenging, which usually results in a lower accuracy in comparison with the genome-guided strategy. Although an increasing number of transcriptome assemblers have been developed to be either genome-guided or de novo, they are all limited by their accuracy in practical applications. As claimed in a recent study [28], even if all the exact exons of a gene are given, they are often unable to assemble the exons into correct isoforms. Therefore, it is imperative to develop novel algorithms for accurate recovery of transcriptomes in eukaryotic species.

In this article, we introduce TransComb, a new genome-guided transcriptome assembler. The basic idea behind TransComb is to subtly extract transcript-representing paths on so-called junction graphs weighted on both their nodes and edges. The weighted junction graph is defined with the nodes representing the edges of a splicing graph and an edge representing a pair of incident edges of the splicing graph. Each node in the junction graph is assigned a weight by the coverage of the corresponding edge of the splicing graph, while each edge in the junction graph is assigned a weight of 0, 1, or 2 defined by novel utilization of the two techniques, bin-packing strategy and paired-end information. It deserves mention that the bin-packing strategy was first developed in our previous de novo assembler BinPacker for combing splicing graphs, but it is subtly employed in TransComb along with the paired-end information to guide the accurate extraction of all the full-length transcripts over the so-called weighted junction graphs mentioned above. Tested on both simulated and real datasets from multiple species, TransComb performs better than all the four compared leading assemblers, including StringTie, Cufflinks, Bayesembler, and Traph on all datasets. For example, TransComb correctly assembled 23 % more full-length transcripts than Bayesembler, the next best assembler, on the human K562 dataset, 12 % more on the human H1 cell dataset, and 13 % more on the mouse dendritic cell dataset. In addition, it runs much faster than Cufflinks and Bayesembler and requires less memory, on average, than all the other assemblers.

## Results and discussion

We first present an overview of our transcriptome recovery model with a detailed description in the “Methods” section, followed by a comparison of TransComb to the state-of-the-art assemblers of the same kind on both simulated and real datasets.

### The TransComb model

TransComb first constructs the splicing graphs ab initio from the alignments of RNA-seq reads to the reference genome. Theoretically, each gene locus is represented by a splicing graph and each exon is represented by a node in the graph and two nodes are connected by a directed edge if and only if there is a splicing junction between them. The coverage of each edge is defined as the number of reads spanning the corresponding junction.

In order to construct more accurate splicing graphs, which would largely improve the performance of an assembler, TransComb utilizes the paired-end reads to repair the fragmented exons due to low expression levels of the gene and further slides a window to correct the wrongly merged exons due to sequencing or mapping errors. Based on the splicing graphs, we develop a rigorous mathematical model to determine the transcripts most likely to be expressed.

**Fig. 1:**
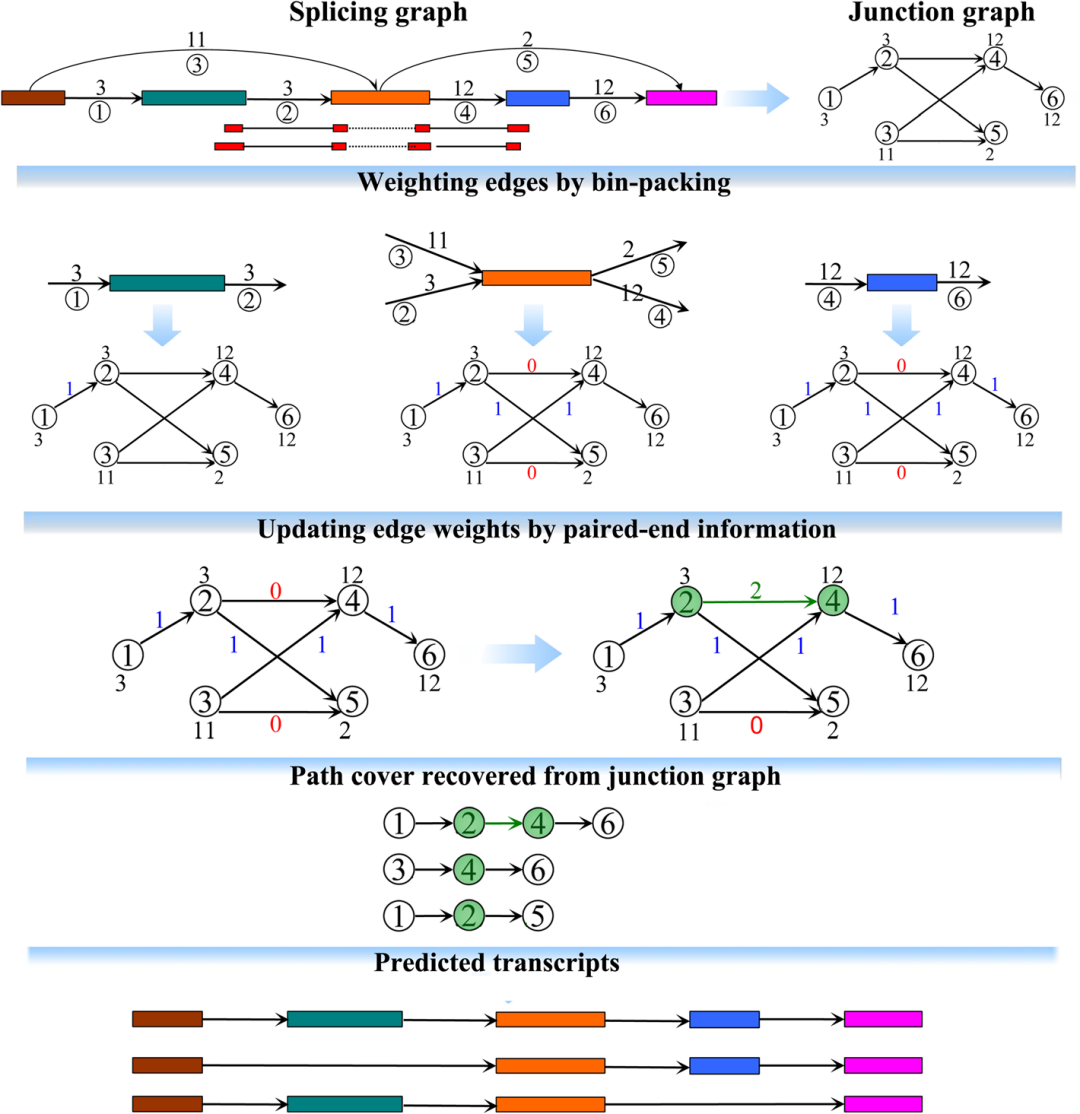
Methodological aspects of TransComb. For the two numbers of an edge in the splicing graph, the number above the edge represents the coverage of the edge and the circled number below represents the index of the edge. The number on each node of the junction graph represents the weight of this node, which is the coverage of the corresponding edge on the splicing graph

Resolving the ambiguities in linking in- and out-splicing junctions at each exon with multiple splicing junctions is the toughest task in the development of assemblers. Most of the existing assemblers suffer from this, resulting in their low assembly accuracies (see an example in Additional file 1: Figure S4). To alleviate this, we designed a new assembly strategy by introducing another directed acyclic graph, termed a *junction graph*, with nodes representing edges in the splicing graph and edges representing two incident edges in the splicing graph. Each node in the junction graph is weighted by the coverage of its corresponding edge in the splicing graph, while each edge is weighted by 0, 1, or 2 defined by employing two techniques, bin-packing strategy and paired-end information. A weight of 0 on an edge is considered as no information supporting this edge being included in any expressed transcript, i.e., it will never exist in any predicted transcript of TransComb. A weight of 1 on an edge means that the information from the bin-packing strategy supports this edge being included in some expressed transcripts with high credibility. In some special cases, the above ambiguities are hard to solve by the bin-packing strategy; e.g., the strategy will have a problem when two transcripts sharing an exon have very similar expression levels. Paired-end information subtly involved in junction graphs could then effectively be used to refine the solution from the bin-packing strategy. Thus, a weight of 2 on an edge implies that the information from paired-end reads supports this edge with high credibility. Benefiting from bin-packing and paired-end information, TransComb can more accurately link in- and out-splicing junctions at each exon (i.e., adjacent nodes in the junction graph) and therefore overcome ambiguities to a great extent. The substantial difference between TransComb and other assemblers lies in their methodological aspects. TransComb simultaneously integrates coverage and paired-end information by jointly applying bin-packing and paired-end techniques to a so-called junction graph rather than working from the splicing graph as the other assemblers do. In addition, we have also developed a novel extension strategy for transcript-representing paths over the junction graphs, by which each predicted path has a very high probability to represent an expressed transcript no matter whether its expression level is low or high (see the “Methods” section and Fig. 1 for more details).

### Performance evaluation

We compared TransComb with four other leading genome-guided assemblers—StringTie, Cufflinks, Bayesembler, and Traph—on both simulated and real datasets using their default and some other parameters on the same server (see Additional file 1 for details of the parameter setups for the compared assemblers). In our experiments, the alignments of RNA-seq reads generated by TopHat2 (see Additional file 1 for download information for the TopHat2 index files) and the reference transcripts downloaded from the Ensembl Genome Browser (see Additional file 1 for download information) were used to evaluate the assemblers on real datasets. A reference transcript is considered to have been correctly recovered if it has the same number of exons as an assembled transcript as well as exactly matched intron boundaries. In this study, Cuffcompare [10] was applied to detect the correctly assembled transcripts. A reference transcript is called a true positive for an assembler if it was exactly recovered by the assembler. Accuracy is measured by recall and precision, where recall is defined as the fraction of true positives out of all expressed reference transcripts in the experiment and precision is defined as the percentage of all assembled transcripts that correctly match a reference transcript. We use recall and precision to evaluate the assembly accuracy of each compared assembler.

#### Performance comparisons on simulated data

We first tested TransComb against the other four assemblers on the simulated dataset used in CIDANE [17] for their performance evaluation (see Additional file 1 for download information for the simulated data). This dataset was generated by Flux Simulator [29] using all known transcripts from the UCSC hg19 gene annotation, which contains approximately 80 million strand-specific RNA-seq paired-end reads of 100-bp length.

Testing the five assemblers, TransComb, StringTie, Cufflinks, Bayesembler, and Traph, with their default settings, TransComb performed the best in transcript recovery, with 10,528 true positives versus 9988 for StringTie, 8281 for Cufflinks, 9835 for Bayesembler, and 8299 for Traph (Additional file 1: Table S1). Broadly, TransComb recovered 5 % more true positives than the next best assembler, StringTie, and the large differences between TransComb and the others can be expected on large real datasets. So it is demonstrated from the comparison results that TransComb achieves higher recall than the other assemblers.

**Fig. 2:**
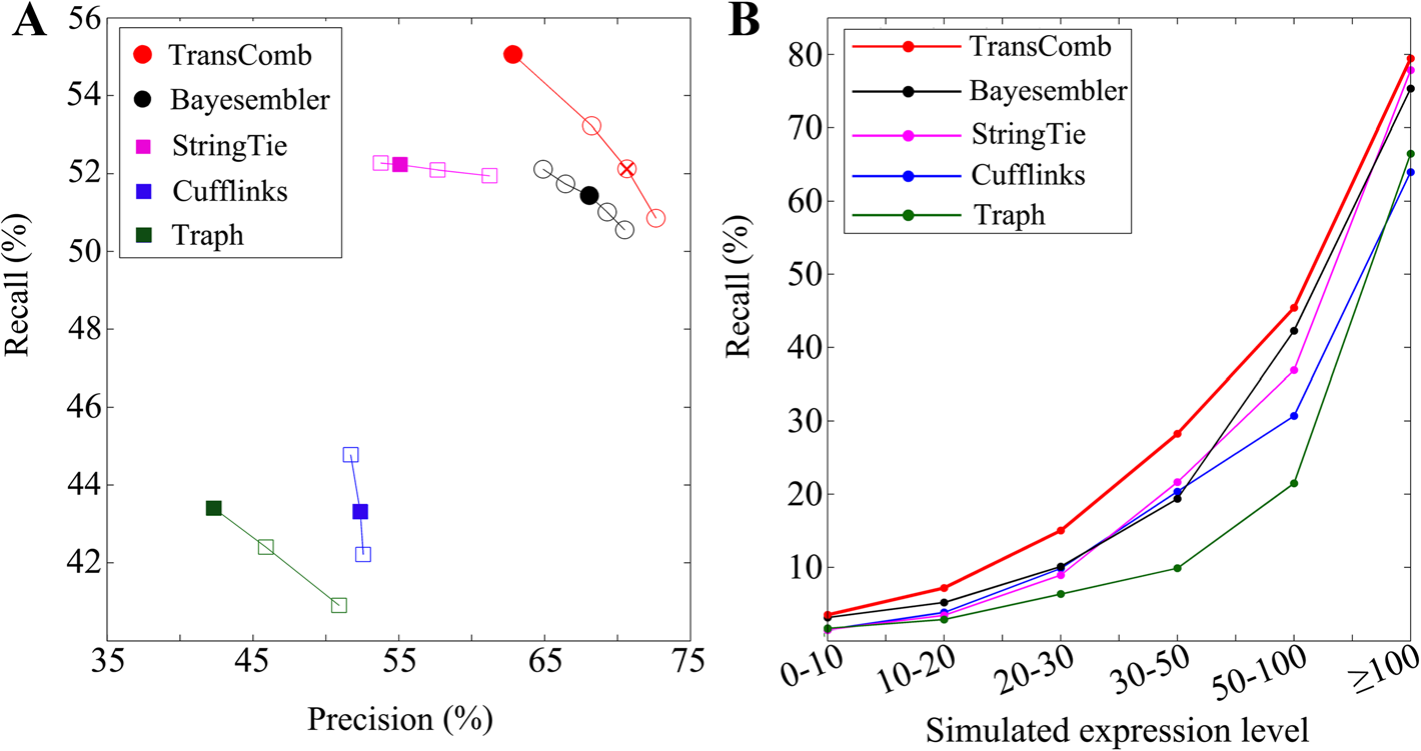
Comparison results on the simulated dataset. **a** Precision and recall values for each assembler. *Solid circles*/*squares* represent the precision and recall values derived using the default settings of the assemblers. *Empty circles*/*squares* represent the precision and recall values using non-default settings of the assemblers. The *crossed circle* represents the precision and recall values of TransComb when filtering its candidates to the same level as Bayesembler. **b** Recall distributions against transcript expression levels

Comparison results for precision show that TransComb reaches 62.83 % versus StringTie with 55.09 %, Cufflinks with 52.36 %, Bayesembler with 68.08 %, and Traph with 42.32 %, where Bayesembler seems to perform the best. However, the better precision of Bayesembler comes from the fact that it filters too many candidates with the default setting, while TransComb does not, making the number of candidates generated by TransComb much larger than that generated by Bayesembler. To make a fair comparison between Bayesembler and TransComb, we filtered the candidates of TransComb using a filtering parameter (see Additional file 1 for details) close in size to that of Bayesembler. The comparison results in Fig. 2a demonstrate that the recall and precision achieved by TransComb are both higher than by those achieved by Bayesembler when filtering their candidates to the same level (see Additional file 1: Table S1 for details of their filtered candidates).

To evaluate the overall performance of the various assemblers, we computed their precision and recall by adjusting their filtering parameters (Fig. 2a; see Additional file 1 for details), showing that TransComb always outperforms the others. So it is concluded that TransComb has the best accuracy amongst the assemblers tested on the simulated dataset.

An ideal assembler should be able to recover more expressed transcripts no matter with low or high expression levels. To evaluate the assemblers in terms of expression levels, we computed their recall distributions against transcripts expression levels in Fig. 2b, showing that TransComb is superior over other compared assemblers across all the expression levels.

#### Performance comparisons on real data

Tests on real data should better evaluate the essence of an assembler because the data have properties that cannot be accurately captured by simulations. In this study, two strand-specific datasets from human K562 cells and H1 cells and one strand-specific dataset from mouse dendritic cells were used for evaluating the performance of the compared assemblers. These three datasets contain 125 million, 41 million, and 53 million paired-end reads, respectively. The human K562 cell and H1 cell RNA-seq datasets were collected from the NCBI Sequence Read Archive (SRA) database with accession codes SRX110318 and SRX082572, respectively. The mouse dendritic cell dataset was also collected from the NCBI SRA database with accession code SRX062280. Unfortunately, Traph does not work on real datasets due to instability, so we only evaluated TransComb against StringTie, Cufflinks, and Bayesembler. Just as presented in [8], it is impossible for us to know all the genuine transcripts encoded in the sample. However, we may consider those assembled as true positives if they correctly match a reference one, or false positives otherwise, without substantially affecting their comparative performance. We thus compared TransComb against the other three assemblers on the three real datasets on this basis and the precision and recall from the simulated dataset were used to evaluate the accuracies of the assemblers on real datasets.

Running the four assemblers with their default settings on the three real RNA-seq datasets, TransComb recovered more true positives than the other three assemblers for each (Additional file 1: Tables S2, S3, and S4). TransComb recovered 12,948, 12,135, and 11,117 true positives for the human K562 and H1 cell and mouse dendritic cell datasets, respectively, versus 10,507, 10,832, and 9874 recovered by the second best assembler, Bayesembler. In comparison with Bayesembler, TransComb recovered 23 % more true positives on human K562 cell data, 12 % more on human H1 cell data, and 13 % more on mouse dendritic cell data. The comparison results thus demonstrate that TransComb achieves the highest recall among all the compared assemblers.

**Fig. 3:**
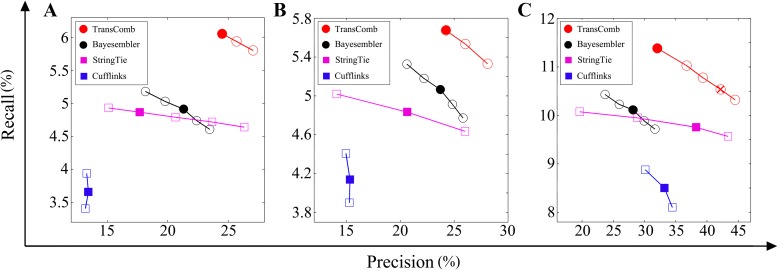
Comparison results on the three real datasets: **a** human k562 cells, **b** human H1 cells, and **c** mouse dendritic cells. *Solid circles*/*squares* represent precision and recall values achieved using default settings. *Empty circles*/*squares* represent precision and recall values achieved using different filtering parameters. The *crossed circle* in **c** represents TransComb’s precision and recall values when filtering its candidates to the same level as StringTie and Cufflinks

As for precision, TransComb achieved 24.5, 24.23, and 32.04 % on the human K562 and H1 cell and mouse dendritic cell data, respectively, versus 17.69, 20.64, and 38.26 % for StringTie, 13.43, 15.33, and 32.95 % for Cufflinks, and 21.3, 23.72, and 28.18 % for Bayesembler. The comparison results show that TransComb performs better than all the other assemblers on the human K562 cell and H1 cell datasets. For the mouse dendritic cell dataset, as stated in the last section, TransComb does not filter its candidates with its default setting while other assemblers all filter their candidates with their default settings, leading to their precision being higher than that of TransComb, with the filtering used by StringTie and Cufflinks filter resulting in the highest precision on the mouse dendritic cell dataset. As with the simulation data, we filtered the candidates of TransComb after setting its filtering parameter (see Additional file 1 for details) to a size close to that of StringTie and Cufflinks. The comparison results in Fig. 3c demonstrate that TransComb also achieved recall and precision levels higher than all the other compared assemblers on the mouse dendritic cell dataset when filtering their candidates to the same level (see Additional file 1: Table S4 for details of their filtered candidates).

As with the simulation, we also compared the precision and recall values when using different filtering parameters (see Additional file 1 for details) on the three real datasets (Fig. 3), showing that TransComb is consistently superior over the other assemblers on all tested real datasets. Therefore, we conclude that TransComb has the best accuracy among all the assemblers tested on both simulated and real datasets.

### Other comparisons on both simulated and real datasets

In addition to the comparisons above, we further compared correctly identified genes and unique true positives (see Additional file 1 for definitions of these criteria) on both simulated and real datasets. The results (Additional file 1) showed that TransComb performs much better than all the compared assemblers according to these criteria, while it is only slightly inferior to StringTie in terms of correctly identified genes on the human H1 cell dataset (see Additional file 1 for further details).

For expression level estimation, we developed a very simple but unusual method which maintains accuracy levels comparable to the other tools but much better than Traph (see Additional file 1 for detailed comparison results). It may provide ideas for scientists interested in developing more powerful estimators.

### Comparison of running time and memory usage

**Fig. 4:**
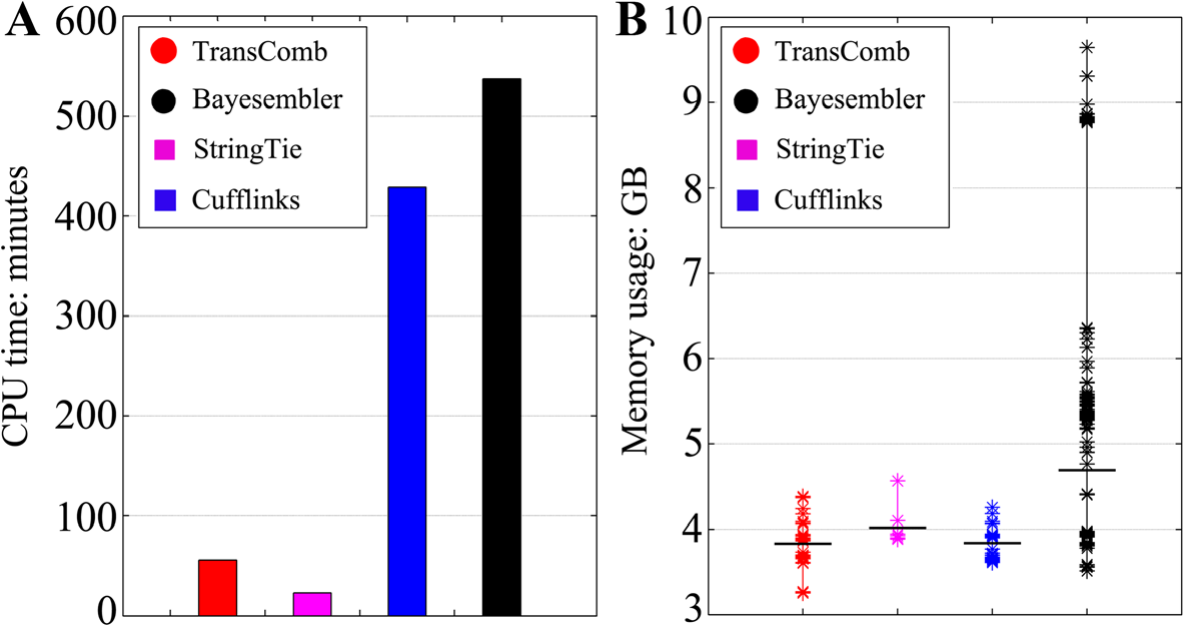
CPU time and memory usage of the four assemblers on the human K562 cell dataset. **a** CPU times of the four assemblers. **b** Memory usage ranges of the four assemblers; the *horizontal black lines* represent the average memory usage for each assembler

We show the running time and memory usage of the four assemblers only for the human K562 cell dataset, which contains the largest number of reads (approximately 125 million reads) among all the tested datasets (Fig. 4; detailed in Additional file 1: Table S10). StringTie used the least CPU time (23 minutes) and TransComb used the second least CPU time (56 minutes), more than seven times faster than Cufflinks (429 minutes) and nine times faster than Bayesembler (537 minutes). StringTie used less time than the other assemblers because it simply applied a greedy algorithm to extend paths in the splicing graphs. In addition, the current version of TransComb was developed to run on one single CPU while StringTie runs on multiple CPUs. We will parallelize TransComb in the near future in order to increase its throughput when multiple CPUs are available.

For memory usage, all four assemblers have a large memory footprint (Additional file 1: Table S10) due to the large RNA-seq dataset to be processed, especially on the highly expressed transcripts. We see from Fig. 4b that TransComb uses less memory than all the others, on average, while Bayesembler suffers the worst memory usage, and StringTie and Cufflinks consume similar memory resources on average.

## Conclusions

We present TransComb, a novel genome-guided computational tool for transcriptome assembly from short RNA-seq reads. Compared to other leading assemblers on both simulated and real datasets, TransComb consistently performs the best. First, in comparison with other genome-guided assemblers, TransComb more accurately identifies the boundaries of gene loci, as well as exons and junction sites within a gene. In lowly expressed genes, some exons may not be fully covered by reads, which results in an exon or a gene being separated into two or more parts. Using paired-end information, TransComb is able to repair some of the broken gene loci and even exons which are not spanned by reads and thus builds more accurate splicing graphs. Second, TransComb is able to recognize pseudo exons, each consisting of two consecutive exons and the intron between them, which may result from wrongly mapping reads to the intron areas, and is capable of distinguishing the actual exons from the pseudo ones. Third, it exploits a combing strategy to search for a path cover on a so-called weighted junction graph, but not a minimum path cover, which has been found to be not always optimal. In contrast, StringTie and Traph were developed based on a network flow model along with a greedy approach for assembling transcripts, which are fragile especially in complicated splicing graphs. Fourth, TransComb subtly integrates paired-end information into its process in order to correct errors caused by either sequencing or mapping. In addition, TransComb is also superior to the assemblers compared here with regard to computational resource usage. It runs the fastest except for StringTie and uses the least memory.

TransComb is comparable to the other assemblers in transcript expression level estimation, just slightly inferior to StringTie but much superior to Traph. It has been observed that exclusively using the seed edges reduces the adverse impact of unexpected errors and biases on the estimation of expression levels of assembled transcripts to some extent. We believe that our combinatorial approach in conjunction with the statistical normalization model will further improve the estimator in the future.

In conclusion, TransComb is essentially distinct from any previous transcriptome assembler. It attempts to achieve a global solution by (1) combing in- and out-edges at each node of splicing graphs; (2) guiding decision at each edge in a so-called weighted junction graph using paired-end reads; and (3) extending the current path based on the weighted junction graph. The software has been developed to be user-friendly. It may play a crucial role in new discoveries in transcriptome studies using RNA-seq, especially in complicated human diseases related to abnormal splicing events and expression levels, such as cancers.

## Methods

### Construction of splicing graphs

Splicing graphs have played a fundamental role in the development of TransComb, which are constructed at each gene locus based on alignments of mapping reads to a reference genome using TopHat2. Aligned reads are first clustered into different gene loci and then the exon–intron boundaries and exon–exon junctions for each gene are derived from junction reads or paired-end reads. Paired-end reads are also used to repair fragmented exons due to low gene expression levels and a sliding window technique is developed to correct the wrongly merged exons due to sequencing or mapping errors. Then, for each gene locus, an exon is represented by a node and two nodes are connected by a directed edge if and only if there is a splicing junction between them. The coverage of each edge is defined as the number of reads which span the corresponding junction. Paired-end information between every two incident edges is also recorded for further use (Additional file 1: Figure S2a). In an ideal case, the splicing graphs would correspond one-to-one to all the (expressed) genes.

Theoretically, a splicing graph captures all possible alternative splicing events, with the nodes in the graph corresponding to continuous regions in the genome that are uninterrupted by any splicing event and directed edges corresponding to splicing junctions between exons. It is worth mentioning that a node in splicing graphs does not necessarily correspond to a true exon in a gene; instead, it may be only a partial exon as illustrated in Additional file 1: Figure S2b.

### Recovery of a set of paths from a splicing graph

Based on the splicing graphs constructed above, TransComb recovers the most likely expressed transcripts by combing the junction edges on each splicing graph. The combing strategy is achieved by the following steps (see Additional file 1: Figure S1 for a flow chart).

#### Construction of junction graphs

To resolve the ambiguities in linking the in- and out-splicing junctions at each exon with multiple splicing junctions, we define another directed acyclic graph *J*, named the *junction graph*, weighted on both its nodes and edges. In *J*, each node *i* represents a junction edge in the splicing graph and two nodes *i* and *j* are connected by a directed edge (*i*, *j*) if and only if *i* and *j* correspond to two incident edges in the splicing graph (as shown in Fig. 1, edges 1 and 2 are incident edges, while edges 1 and 3 are not). Each node is weighted by the coverage of its corresponding edge in the splicing graph, while each edge is suitably weighted based on the novel use of a bin-packing strategy and paired-end information, described in the following two sections.

#### Weighting edges in junction graphs by the bin-packing strategy

To decide whether or not two consecutive nodes in a junction graph come from a common transcript, we developed a rule to assign to each edge in the junction graph a weight of either 0 or 1, with 1 meaning that the two nodes of the edge would possibly come from a single transcript and 0 meaning they will never do so. The rule was designed by using a variant of the traditional bin-packing model which has been well studied in the field of operations research.

For each node *v* with both in-edges and out-edges in a splicing graph, it is required to optimally link between in-edges and out-edges such that two linked edges in the splicing graph which correspond to two nodes in the junction graph come from a common transcript with a higher probability of being correct. Assume that node *v* has *n* in-edges and *m* out-edges with *n* ≥ *m*, then TransComb identifies each in-edge as an item with its size *s*_*j*_ being the coverage of the in-edge and each out-edge as a bin with its capacity *c*_*i*_ being the coverage of the out-edge. The optimum link between in-edges and out-edges is then achieved by optimally packing the items (in-edges) into the bins (out-edges). To solve this optimal packing problem, we define a binary variable *x*_*ij*_, with *x*_*ij*_ = 1 if item *j* is packed into bin *i*, and 0 otherwise. This problem can be reduced to find an optimum solution {*x*_*ij*_} to minimize the following quadratic function on the condition that each item goes into one and only one bin and each bin receives at least one item:

1 $$ \begin{array}{l}z={\displaystyle \sum_{i=1,\dots, m}{\left({c}_i-{\displaystyle \sum_{j=1,\dots, n}{s}_j{x}_{ij}}\right)}^2}\\ {}\begin{array}{cc}\hfill s.t.\hfill & \hfill \left\{\begin{array}{c}\hfill \begin{array}{cc}\hfill {\displaystyle \sum_{i=1,\dots, m}{x}_{ij}=1}\hfill & \hfill \forall j=1,\dots, n\hfill \end{array}\hfill \\ {}\hfill \begin{array}{cc}\hfill {\displaystyle \sum_{j=1,\dots, n}{x}_{ij}\ge 1}\hfill & \hfill \forall i=1,\dots, m\hfill \end{array}\hfill \\ {}\hfill {x}_{ij}\in \left\{0,1\right\}\hfill \end{array}\right.\hfill \end{array}\end{array} $$

The same formula was used in BinPacker, a de novo transcriptome assembler. As stated in BinPacker, this quadratic programming can be quickly solved. Based on the solution {*x*_*ij*_} of Eq. 1, we assign to each edge connecting nodes *i* and *j* in the junction graph the weight *w*(*i*, *j*) = *x*_*ij*_. Intuitively, two nodes connected by an edge of weight 1 would come from a common transcript with a higher probability of correctness, while an edge of weight 0 is considered as having no information supporting this edge being included in any expressed transcript and so it will never exist in any predicted transcript.

#### Updating edge weights in junction graphs using paired-end information

All paired-end information between two incident edges in each splicing graph was recorded, as were constructed splicing graphs. We are guided by this kind of paired-end information to update the edge weights of the corresponding junction graph as follows. For each two incident edges *i* and *j* in a splicing graph, if at least two mate pairs supporting these two edges are recorded (see Additional file 1: Figure S2A for details), then the weight of the edge connecting these two corresponding nodes in the junction graph is replaced by 2. Therefore, two nodes connected by an edge of weight 2 in the junction graph are most likely to come from a common transcript.

Clearly, the weighted junction graph defined above will guide us to more accurately extract the full-length transcripts. Based on the junction graph, TransComb is able to more accurately link the in- and out-splicing junctions at each exon. The substantial difference between TransComb and the other assemblers is that TransComb works on the junction graphs rather than the splicing graphs on which the others do. On the other hand, the extension strategy developed for TransComb is another unique feature distinguishing it from the other assemblers.

#### Recovery of full-length transcripts from weighted junction graphs

To extract a transcript-representing path in the junction graph, we first choose a node with the largest weight as the seed node, which is excluded from any predicted path. The seed node is then extended to one of its right neighboring nodes according to the weights of their corresponding edges. An edge of weight 2 has the first priority and an edge of weight 1 has the second priority for extension. We keep extending towards the right until we encounter a node without out-going edges. Similar extension is done towards the left and then a transcript-representing path is predicted. This procedure is repeated until all the nodes in the junction graph have been included in predicted paths (see Additional file 1 for detailed description of the path extension procedure via pseudo code).

It is clear that in each extension towards left or right, TransComb always extends the current path to the neighboring node supported by either bin-packing or paired-end information and so each predicted path has a very high probability to represent an expressed transcript no matter whether its expression level is low or high.

### Estimation of expression levels of the recovered transcripts

Based only on the seed nodes used during the path extension from the junction graph, we developed a very simple but unusual method for expression level estimation (see Additional file 1 for a detailed description of this method).

## Additional file

Additional file 1: Supplementary materials. This file contains details of splicing graph construction, additional comparisons with other methods, and supplementary figures and tables. (PDF 7353 kb)

## Abbreviations

NCBI: National Center for Biotechnology Information
RNA-seq: RNA sequencing
SRA: Sequence Read Archive
