# Supplementary material for: TransComb: genome-guided transcriptome assembly via combing junctions in splicing graphs
Source: Genome Biol. 2016 Oct 19;17:213. doi: 10.1186/s13059-016-1074-1 (PMC5069867; doi:10.1186/s13059-016-1074-1)
Supplement: Supplementary file 1 — Supplementary materials. This file contains details of splicing graph construction, additional comparisons with other methods, and supplementary figures and tables. (PDF 7353 kb) [file 13059_2016_1074_MOESM1_ESM.pdf]

Supplementary Materials

TransComb: genome-guided transcriptome assembly via combing junctions in splicing graphs

Juntao Liu[1,2,†], Ting Yu[1,†], Tao Jiang[2,3,4,*], Guojun Li[1,†,*]

1. Supplementary Notes

1.1 Parameter setup for the compared assemblers

The parameters of each genome-guided assembler were set up as their defaults with the same input file produced by Tophat2. All the assemblers were tested on a server with 96GB of RAM. In order to adjust the default filtering parameter of each assembler, we use the following settings. For TransComb (version 1.0), we set –f to be 2, 3 and 4 on simulated dataset; on human K562-cells dataset, this parameter was set to be 1 and 2; on human H1-cells dataset, it was set to be 1 and 2; on mouse dendritic-cells dataset, it was set to be 2, 3, 4 and 5. For StringTie (version 1.1.2), we set –c to be 1.5, 3.5 and 4.5 on simulated dataset; on human K562-cells dataset, this parameter was set to be 1.5, 3.5, 4.5 and 5.5; on human H1-cells dataset, it was set to be 1.5 and 3.5; on mouse dendritic-cells dataset, it was set to be 0, 1.5 and 3.5. For Cufflinks (version 2.1.1), we set –F to be 0.05 and 0.15 on simulated dataset and three real datasets. For Bayesembler (version 1.2.0), we set –c to be 0.3, 0.4, 0.6 and 0.7 on simulated dataset and three real datasets. For Traph (version 0.5), we set --expressionratio to be 0.1 and 0.2 on simulated dataset.

1.2 Tophat2 index files downloaded for real datasets.

Index files are needed for tophat2 mapping, so we downloaded the human index files from ftp://igenome:G3nom3s4u@ussd-ftp.illumina.com/Homo_sapiens/Ensembl/GRCh37/Homo_sapiens_Ensembl_GRCh37.tar.gz

And the mouse index files are downloaded from ftp://igenome:G3nom3s4u@ussd-ftp.illumina.com/Mus_musculus/Ensembl/NCBIM37/Mus_musculus_Ensembl_NCBIM37.tar.gz

1.3 Reference transcripts downloaded for real datasets

In order to evaluate the performances of the assemblers on real datasets, we downloaded the reference transcripts of human and mouse from the Ensemble Genome Browser. The human reference transcripts are downloaded from

http://ftp.ensembl.org/pub/release-73/gtf/homo_sapiens/Homo_sapiens.GRCh37.73.gtf.gz

and the mouse reference transcripts from

http://ftp.ensembl.org/pub/release-67/gtf/mus_musculus/Mus_musculus.NCBIM37.67.gtf.gz

1.4 Simulated data downloading websites

This simulated data was used in CIDANE. The Tophat2 alignment file can be downloaded from

https://uchicago.app.box.com/v/CIDANE-FluxSim/1/5654933313

and the ground truth of the simulated data from

https://uchicago.app.box.com/s/fj5g566nar2evwjl3qpbtrgaeuftt12x/1/8607781925

2. Supplementary Methods

2.1 Construction of splicing graphs

The splicing graphs are constructed based on mapping the RNA-seq reads to a reference genome by Tophat2. Generally speaking, if the reference genome is well resolved and most RNA-seq reads are of high sequencing quality, the mapping results of Tophat2 could be highly reliable. Then TransComb could effectively and efficiently construct splicing graphs based on the mapped results detailed as follows.

1) Clustering reads into gene loci

Since the mapped reads in the output bam file of Tophat2 have been sorted according to their mapping positions from 5’ to 3’, one can easily cluster the mapped reads into their corresponding gene loci. To do so, by *Max_Pos* we denote the most right position of the current cluster of mapped reads in the reference genome, which is initialized by the 3’ end of the first read. TransComb then determines the end-positions of the successive mapped read in

the reference genome, which are represented respectively by *5'_end* and *3'_end*. If *5'_end* is not larger than *Max_Pos*, TransComb clusters this read into the current gene locus, and updates *Max_Pos* by *3' _end* if and only if *3' _end* is larger than *Max_Pos*. TransComb repeats the procedure until it encounters a read with its *5'_end* larger than *Max_Pos*, with which it resumes next gene locus, until all reads exhausted.

2) Identification of exons and splicing junctions in each gene locus

For each gene locus constructed above, we use all the reads clustered into this locus to detect exons and splicing junctions in this gene. To do so, we denote by *Gene_Exon* a zero vector with the same length as this gene locus, with its components corresponding to the positions of this locus, and by *Gene_Junction* a 3-column matrix to represent splicing junctions in this locus. For the matrix *Gene_Junction*, its first two columns store the start and end positions of corresponding junction, and the third column stores the number of reads supporting the junction. Then for each read clustered into this locus, if it is not a junction read, we then add $1/n$ to each aligned components of *Gene_Exon*, where n represents the number of different mappings of this read; otherwise, i.e. this read is a junction read, TransComb updates the matrix *Gene_Junction* by adding a row consisting of start and end positions of the junction as well as the number $1/n$, if this junction has not been detected yet. However, if the junction has already existed, TransComb directly updates the matrix *Gene_Junction* by adding $1/n$ to the number of reads supporting this junction. TransComb Repeats the procedure until all mapped reads belonging to this locus have been exhausted. Then the zero components of *Gene_Exon* constitute the introns and the others the exons with their values representing the coverage of reads supporting this position.

3) Updating of gene loci

We remove those junctions from *Gene_Junction* of their coverage less than 1 because such junctions are not reliable. Notice that a gene locus may be split into two or more gene loci after the updating operation and the following analysis is subjected to updated gene loci.

4) Updating exons and junctions in a gene locus

Biologically, most introns are actually quite long in mammalian genomes. Therefore, for each

two adjacent exons in a gene locus, TransComb combines them into one if either their distance is no more than a prespecified number (the default is set to 100 bp) or there are two or more paired-end reads supporting these two exons (see Figure S2A) and meanwhile the distance between them is more than 100 bp but less than the value of average pair-gap length (the default is set to 200 bp).

5) Updating exons and junctions between two gene loci

For two adjacent gene loci, TransComb merges them into one if either the distance between the two gene loci is no more than a prespecified number (the default is set to 100 bp), or they are supported by at least two paired-end reads if the distance between them is more than 100 bp but less than the value of average pair-gap length (the default is set to 200 bp).

6) Dividing wrongly merged exons

Some reads may be wrongly mapped to intron areas, leading to wrongly merged exons. TransComb attempts to solve this problem by dividing one exon into two exons through sliding a window as follows. For an exon with length *L* longer than 200, TransComb computes the average coverage of the first 50 bp, denoted by *Ave-left*, and that of the last 50 bp, denoted by *Ave-right*. Then it slides a 50-bp-length widow from left to right by one bp at a time (See Figure S3) and computes the average coverage of each sliding window, and finally picks the minimum one, denoted by *Ave-min*. If *Ave-min* is no more than 25% of the smaller one of *Ave-left* and *Ave-right*, then this exon is divided into two exons from the middle of the corresponding window of *Ave-min*.

7) Generation of splicing graphs

For each gene locus, an exon recorded in *Gene_Exon* is represented by a node and any two nodes are connected by a directed edge from 5' end to 3' end if and only if there is a splicing junction recorded in *Gene_Junction* supporting it. The coverage of each node is computed as the average coverage of each base in the corresponding exon recorded in *Gene_Exon* and the coverage of each edge is recorded in the third column of *Gene_Junction*. By *Pair_Edges* we denote a 2-column matrix to record pair supporting information between two edges in the splicing graph, which is useful and important in extracting paths from the splicing graphs. For

each two edges in a splicing graph, if they are supported by at least two mate pairs (see Figure S2A), *Pair_Edges* will be updated by adding a row with its two entries being the indexes of these two edges.

2.2 Recovery of full-length transcripts from weighted junction graphs

To do so, by *Nodes_Unused* we denote the dynamic set of nodes in the junction graph $J = (V(J), E(J))$ that have not been covered by assembled paths and by *filter* a parameter for determination. Initially, *Nodes_Unused* is set to $V(J)$. Each transcript-representing path in a splicing graph is extracted by recurrently extending a current path in the corresponding weighted junction graph originating from a seed node in *Nodes_Unused.* In the procedure, by n_l and n_r we respectively denote the left and right ends of the current path which is being extended in the junction graph. By N_L (resp. N_R) we denote the set of left/in (resp. right/out) neighbors of n_l (resp. n_r) in the junction graph, i.e. $N_L = \{v \in V(J) \mid (v, n_l) \in E(J)\}$ (resp. $N_R = \{v \in V(J) \mid (n_r, v) \in E(J)\}$). We further set $N_L^1 = \{v \in N_L \mid w(v, n_l) = 1\}$, $N_L^2 = \{v \in N_L \mid w(v, n_l) = 2\}$; $N_R^1 = \{v \in N_R \mid w(n_r, v) = 1\}$, $N_R^2 = \{v \in N_R \mid w(n_r, v) = 2\}$. Then TransComb iteratively extracts transcript-representing paths in a splicing graph through the operations on the corresponding junction graph that are pseudo-coded by the following steps.

Step 1. If *Nodes_Unu*sed $\neq \emptyset$, TransComb chooses from *Nodes_Unu*sed a seed node v of largest weight *cov_max* ($n_l = n_r = v$ at the moment); or else, it terminates. If *cov_max* < *filter*, it removes from *Nodes_Unu*sed the node v, returns to Step 1; or else, goes to Step 2;

Step 2. If $N_L^2 \neq \emptyset$, TransComb extends from n_l to a node n_l' in N_L^2 of maximum weight, resets $n_l = n_l'$, updates the current sets N_L, N_L^2 and N_L^1 accordingly, and returns to Step 2; or else

if $N_L^1 \neq \emptyset$, TransComb extends from n_l to a node n_l' in N_L^1 of maximum weight, resets $n_l = n_l'$, updates the current sets N_L, N_L^2 and N_L^1 accordingly, and returns to Step 2; or else, goes to Step 3;

Step 3. If $N_R^{-1} \neq \emptyset$, TransComb extends from n_r to a node n_r' in N_R^{-1} of maximum weight, resets $n_r = n_r'$, updates the current sets N_R, N_R^{-1} and N_R^{+1} accordingly, and returns

to Step 3; or else

if $N_R^1 \neq \emptyset$, TransComb extends from n_r to a node n_r' in N_R^1 of maximum weight, resets $n_r = n_r'$, updates the current sets N_R, N_R^2 and N_R^1 accordingly, and returns to Step 3; or else, goes to Step 4;

Step 4. TransComb removes from *Nodes_Unused* the nodes of the path obtained by Steps 1, 2 and 3, and returns to Step 1.

The default value of *filter* is set to 0 by TransComb, implying that it will not terminate until all the nodes in the junction graph have been covered by the assembled paths. It is worth mentioning that nodes removed from *Nodes_Unused* can still be used for extension of other paths.

2.3 Estimation of expression levels of the recovered transcripts

Intuitively, expression level of each recovered transcript would have been estimated by allocating the coverage of each edge to the expressed transcripts if the expressed transcripts were uniformly sampled and fragmented. However, the fragmentation and sequencing process may cause a lot of biases and even errors, and moreover, the process of mapping RNA-seq reads to a reference genome may also bring in many unexpected errors. On the other hand, the assembled transcripts are not necessarily the true expressed ones. So it may not be a good idea to estimate the expression levels for assembled transcripts based on allocation of the coverage of all the edges. To diminish the impact of the errors and biases mentioned above on estimating expression levels for the assembled transcripts, we would prefer to rely on those edges used as seed nodes in the corresponding junction graph during the path extraction procedure rather than all of them, and from now on such edges are called seed edges. Seed edges would be quite reliable because each of them has the highest coverage in the unused nodes during the process of extracting a set of paths from a junction graph. We found that the edges with higher (lower) coverage are less (more) impacted by mapping and sequencing errors. In addition, all assembled transcripts must be covered by these seed edges. Therefore, the expression levels would be better estimated by allocating the coverage only for a few of seed edges to the assembled transcripts. Notice that assembled transcripts one-to-one

correspond to those seed nodes used in the extraction procedure. By $E = \{e_1, e_2, \ldots, e_n\}$ we denote the set of n seed edges, and $T = \{t_1, t_2, \ldots, t_n\}$ the set of n assembled transcripts. It can be achieved by solving the following quadratic programming:

$$\min f = \sum_{i=1,\ldots,n} (w_i - \sum_{e_i \in t_j, j=1,\ldots,n} x_j)^2$$
$$s.t. \quad x_j \geq 0 \quad j = 1,\ldots,n$$

where w_i ($i = 1, 2, \ldots, n$) represents the coverage of the seed edge e_i, and x_j ($j = 1, 2, \ldots, n$) the expression level of transcript t_j.

3. Supplementary Results

3.1. Comparison of correctly identified genes

A reference gene is considered to be correctly identified if at least one isoform in the gene is exactly matched by a predicted transcript in a predicted gene. On the simulated dataset, the comparison results showed that TransComb correctly identified 8948 genes, vs. StringTie 8734, Cufflinks 8281, Traph 7131, and Bayesembler 7814 (see Figure S5A). On the three real datasets, TransComb still performed the best among the other compared assemblers in terms of correctly identified genes while only slightly inferior to StringTie on human H1-cells dataset. For example, the numbers of correctly identified genes of TransComb on human K562-cells, human H1-cells and mouse datasets are respectively 8610, 8762 and 8540, vs. StringTie 8591, 8780 and 8411, Cufflinks 6649, 7532 and 4953, and Bayesembler 8593, 8710 and 8471 (see Figure S6 A, B and C).

3.2. Comparison of detecting unique true positives

To compare two assemblers in terms of their ability of correctly recovering novel isoforms, we define another true positive, termed unique true positive, which is a reference transcript recovered by exactly one of the two compared assemblers. Obviously, the more unique true positives an assembler recovers, the better it performs than others.

We compared the unique true positives between TransComb and each of the other four compared assemblers on the simulated dataset, and found that TransComb assembles much

more unique true positives compared to each of them. For example, the unique true positives are 1385 and 845 between TransComb and StringTie, 2855 and 608 between TransComb and Cufflinks, 2889 and 660 between TransComb and Traph, and 1787 and 1094 between TransComb and Bayesembler (see Figure S5B and Table S5). Overall, TransComb substantially outperforms all the compared assemblers in terms of their unique true positives even in the case where it assembles fewer candidates than others.

The competition in unique true positives between two assemblers was also carried out on real datasets. The competition results show that TransComb assembled much more unique true positives than each of the compared assemblers (see Figure S7 and Table S6, S7 and S8). Overall, we conclude that TransComb consistently and overwhelmingly outperforms each of the compared assemblers in terms of unique true positives on both synthetic and real datasets.

3.3 Results of processing wrongly merged exons

If some reads were wrongly mapped to intron areas, then this intron and its two neighboring exons may be wrongly assembled into a single exon, which could result in two transcripts merged into one. TransComb attempts to solve this problem by dividing the whole exon into two as mentioned in the supplementary methods section. Successfully handling this problem significantly improves the performance of TransComb, especially in real datasets. On the simulated dataset, RNA-seq reads are indeed from the reference genome, which will be used as the mapping template, so the error mapping rates may be quite lower than on real datasets, which may contain mutations, indels, individual differences and some other unknown factors resulting from the sequencing process. So much fewer exons in simulated datasets will be divided than in real datasets. For example on the simulated dataset, the number of exons divided is 52. While in real datasets, the numbers are 5739, 3760 and 2096 on human K562-cells, human H1-cells and the mouse datasets, respectively. Then we computed the number of assembled true positives resulting from those divided exons on both simulated and real datasets. On the simulated dataset, the number of such true positives is 32 (Figure S5C and Table S9), while on the real datasets, the numbers are 1627, 1079 and 650 (Figure S8 and Table S9) on human K562-cells, human H1-cells and the mouse datasets, respectively. We can

see that the exon-splitting tip indeed makes a great contribution to the performance of TransComb, especially on real dataset.

3.4 Comparison of expression level estimations

After assembling the RNA-seq reads into transcripts, another challenging task is to estimate the expression abundance of each assembled transcript in original cells. To evaluate the estimators, we compared the estimated abundances with the genuine ones for those correctly assembled transcripts only by all the five assemblers. The distribution of the simulated transcripts against their expression levels is shown in Figure S9A. For each estimator, we used the Spearman correlation coefficient between the simulated and estimated FPKMs to measure the estimators. As shown in Figure S9, the correlation coefficient of TransComb is 0.97, against 0.98 of StringTie, 0.97 of both Bayesembler and Cufflinks, and 0.87 of Traph. The comparison results showed that it reaches comparable accuracy level to the existing tools of same kind, e.g. StringTie, Cufflinks and Bayesembler, while it is much better than Traph. From the design of our method and then its performance, it seems that the expression levels of encoded transcripts would be mainly determined by several key junction edges in the splicing graph. As this method is really unusual and very simple, it may hopefully provide a hint for scientists who are interested in developing more powerful estimator.

4. Supplementary Figures

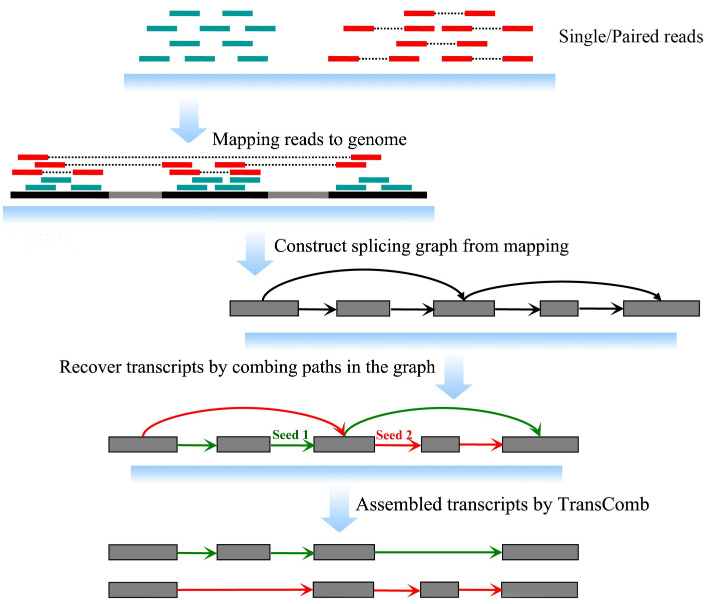

Figure S1. Transcriptome assembly pipeline for TransComb.

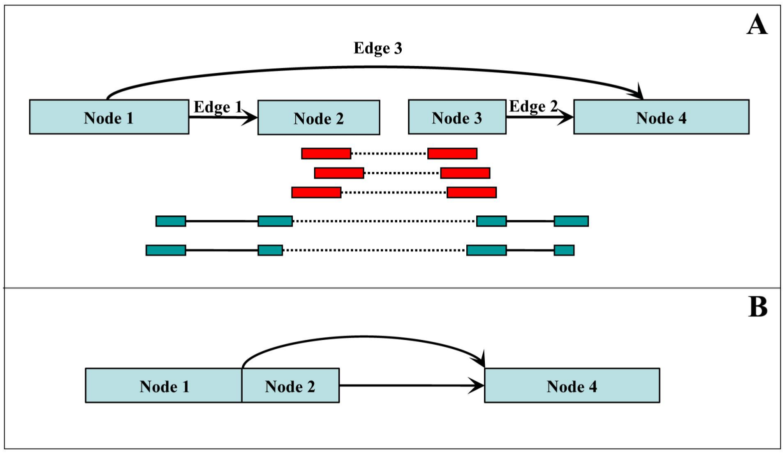

Figure S2. Examples for paired end reads supporting information and partial exons. (A) Paired end reads colored red indicates that Node 2 and Node 3 are supported by 3 paired end reads. Paired end reads colored green indicates that Edge 1 and Edge 2 are supported by 2 paired end reads. (B) An example for showing partial exons.

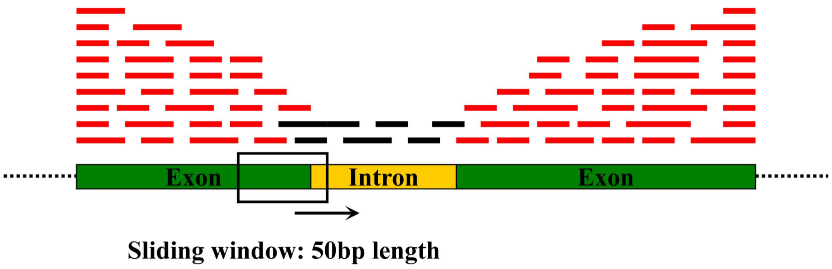

Figure S3. Wrongly merged exons resulting from wrongly mapping reads (black bars representing

reads wrongly mapped to intron areas) and TransComb attempting to divide these merged exons by sliding windows.

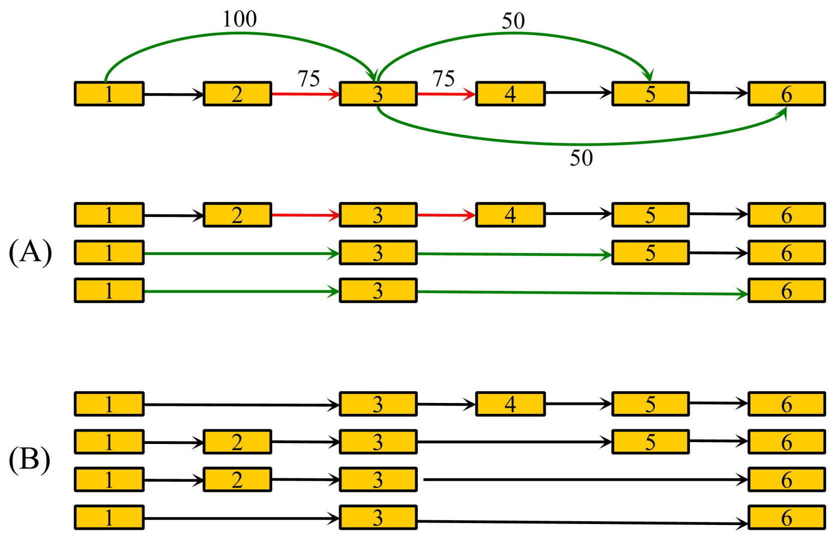

Figure S4. A splicing graph containing ambiguity in exon 3 illustrating those greedy algorithms for searching for paths like StringTie may easily fail. The transcripts expressed in the splicing graph are actually the path-cover in (A) predicted by TransComb. If we use a greedy algorithm such as StringTie, the predicted transcripts may be the paths in (B).

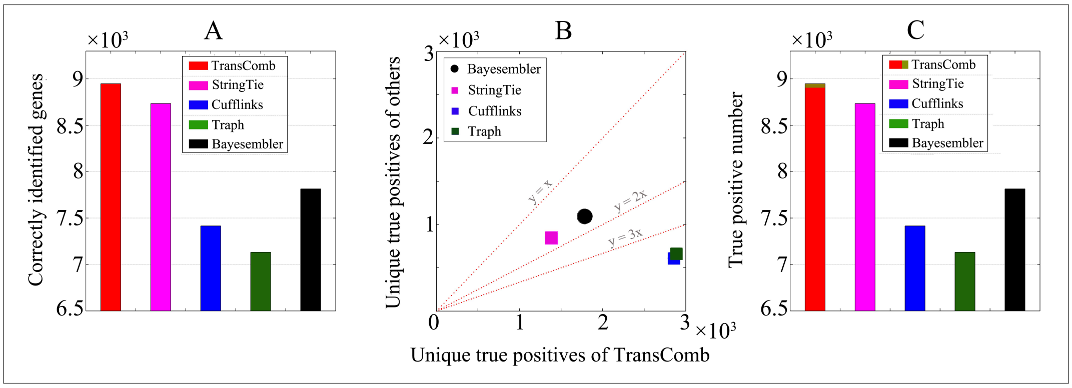

Figure S5. Corresponding results on simulated dataset. (A) Correctly identified genes of the five assemblers. (B) Pairwise unique true positives between TransComb and the other assemblers. For each assembler in this figure, the Y-coordinate represents its unique true positives, while the X-coordinate represents the unique true positives of TransComb. (C) Comparison of assembled true positives illustrating the parts of TransComb resulting from divided exons on simulated datasets. The red bar (assembled true positives from non-divided exons) plus the brown bar (assembled true positives from divided exons) constitute the entire assembled true positives of TransComb.

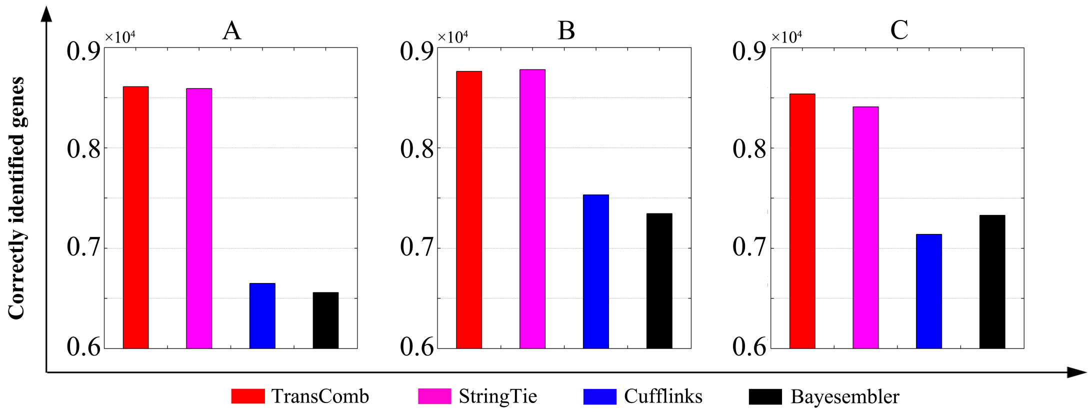

Figure S6. Correctly identified genes of the five assemblers on (A) human K562-cells dataset. (B) human H1-cells dataset. (C) mouse dendritic-cells dataset.

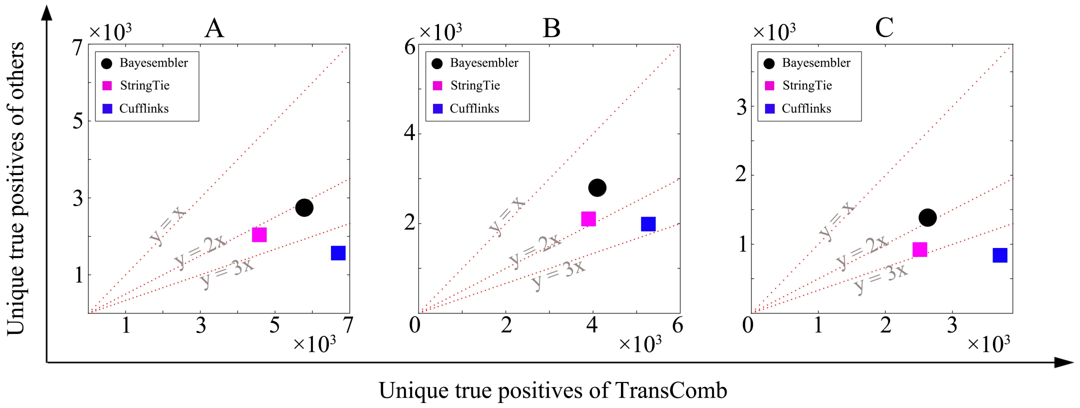

Figure S7. Pairwise unique true positives between TransComb and the other three assemblers on (A) human K562-cells, (B) human H1-cells and (C) mouse dataset. For each assembler in this figure, the Y-coordinate represents its unique true positives, while the X-coordinate represents the unique true positives of TransComb.

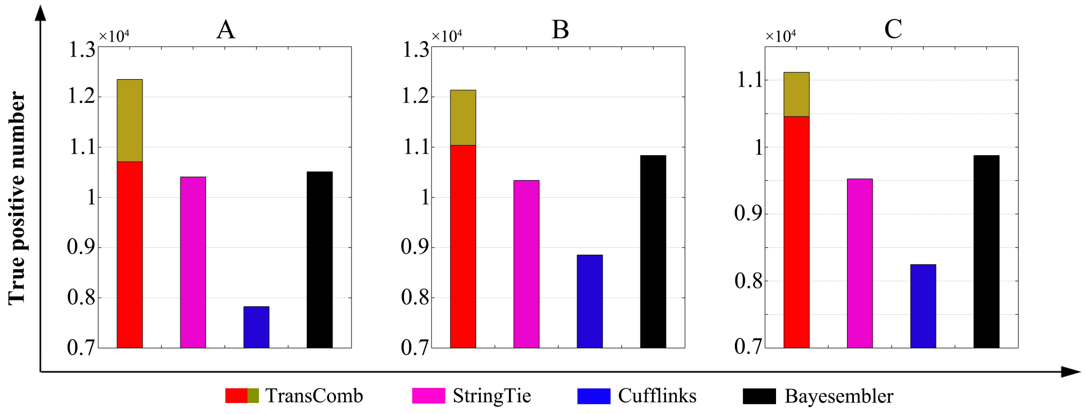

Figure S8. Comparison of assembled true positives illustrating the parts of TransComb resulting from divided exons on real datasets. The red bar (assembled true positives from non-divided exons) plus the

brown bar (assembled true positives from divided exons) constitute the entire assembled true positives of TransComb. (A) Assembled true positives of the four compared assemblers on human K562-cells dataset. (B) Assembled true positives of the four compared assemblers on human H1-cells dataset. (C) Assembled true positives of the four compared assemblers on mouse dataset.

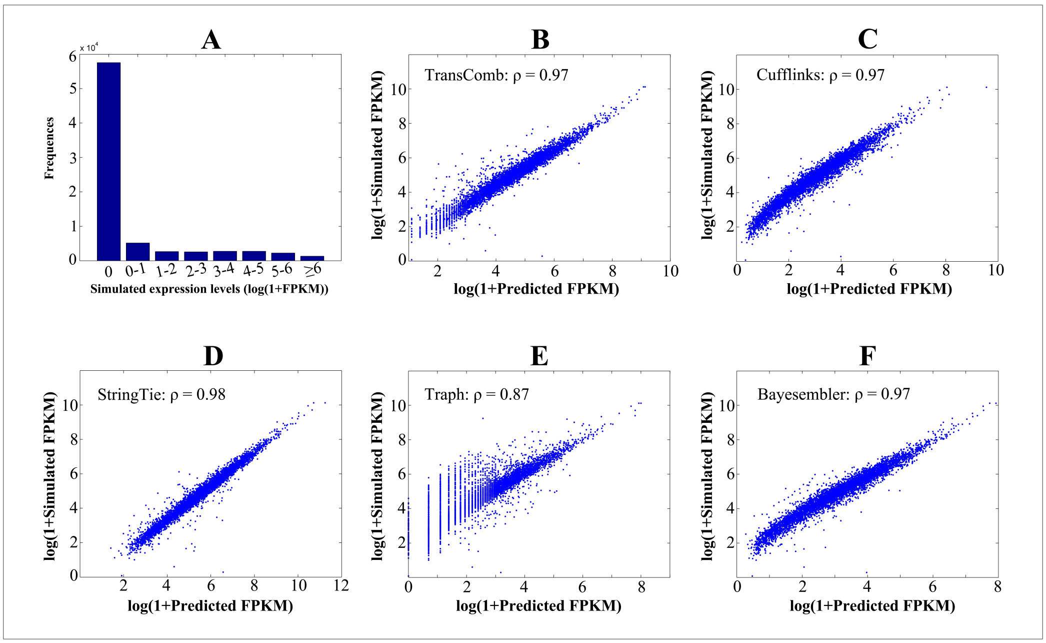

Figure S9. The distribution of simulated isoform expression levels and Correlation between simulated (y-axis) and estimated (x-axis) expression levels on simulated dataset using only transcripts that were correctly assembled by the five assemblers. ρ represents the Spearman correlation coefficient between simulated and estimated FPKM values..

5. Supplementary Tables

Table S1 Candidates and true positives of the assemblers on the simulated dataset.

Assemblers	Filter settings	Candidates	True positives
TransComb	default	16755	10528
	2	14919	10178
	3	14111	9968
	4	13389	9725
StringTie	default	18131	9988
	1.5	18591	9996
	3.5	17274	9962
	4.5	16222	9933
Cufflinks	default	15816	8281

	0.05	16561	8562
	0.15	15355	8071
Bayesembler	default	14447	9835
	0.3	15357	9966
	0.4	14893	9893
	0.6	14080	9755
	0.7	13714	9667
Traph	default	19608	8299
	0.1	17669	8108
	0.2	15361	7821

Table S2 Candidates and true positives of the assemblers on human K562-cells dataset.

Assemblers	Filter settings	Candidates	True positives
TransComb	default	52855	12948
	1	49437	12700
	2	45863	12409
StringTie	default	58818	10407
	1.5	69806	10545
	3.5	49616	10243
	4.5	42645	10092
	5.5	37690	9923
Cufflinks	default	58232	7823
	0.05	63357	8422
	0.15	55263	7286
Bayesembler	default	49320	10507
	0.3	61052	11079
	0.4	54390	10757
	0.6	45271	10135
	0.7	41983	9853

Table S3 Candidates and True positives of the assemblers on human H1-cells dataset.

Assemblers	Filter settings	Candidates	True positives
TransComb	default	50077	12135
	1	45461	11835
	2	40572	11400
StringTie	default	50081	10336
	1.5	76141	10734
	3.5	38108	9912
Cufflinks	default	57757	8852
	0.05	63005	9425

	0.15	54564	8338
Bayesembler	default	45671	10832
	0.3	55271	11392
	0.4	49906	11077
	0.6	42324	10509
	0.7	39525	10205

Table S4 Candidates and True positives of the assemblers on mouse dataset.

Assemblers	Filter settings	Candidates	True positives
TransComb	default	34693	11117
	2	29320	10769
	3	26756	10524
	4	24394	10289
	5	22654	10079
StringTie	default	24896	9525
	0	50134	9838
	1.5	33634	9714
	3.5	21546	9343
Cufflinks	default	25025	8245
	0.05	28786	8669
	0.15	22953	7910
Bayesembler	default	35033	9874
	0.3	43013	10186
	0.4	38476	9987
	0.6	32241	9655
	0.7	29959	9488

Table S5 Unique true positives and common detected reference transcripts between TransComb and the other assemblers on the simulated dataset.

Assemblers	Detected by both	Unique true positives
TransComb	9143	1385
StringTie		845
TransComb	7673	2855
Cufflinks		608
TransComb	8741	1787
Bayesembler		1094
TransComb	7639	2889
Traph		660

Table S6 Unique true positives and common detected reference transcripts between TransComb and the other assemblers on human K562-cells dataset.

Assemblers	Detected by both	Unique true positives
TransComb	8367	4581
StringTie		2040
TransComb	6255	6693
Cufflinks		1568
TransComb	7762	5186
Bayesembler		2745

Table S7 Unique true positives and common detected reference transcripts between TransComb and the other assemblers on human H1-cells dataset.

Assemblers	Detected by both	Unique true positives
TransComb	8236	3899
StringTie		2100
TransComb	6863	5272
Cufflinks		1989
TransComb	8034	4101
Bayesembler		2798

Table S8 Unique true positives and common detected reference transcripts between TransComb and the other assemblers on mouse dataset.

Assemblers	Detected by both	Unique true positives
TransComb	8602	2515
StringTie		923
TransComb	7406	3711
Cufflinks		839
TransComb	8487	2630
Bayesembler		1387

Table S9 Number of exons, devided exons and correctly assembled transcript based on divided exons in the simulated dataset and three real datasets.

Datasets	Exon counts	Divided exon counts	Correctly assembled transcript based on divided exons
Simulation	106273	52	232
Human K562-cells	418116	5739	1627

Human H1-cells	670358	3760	1079
Mouse	275707	2096	650

Table S10 Running time and memory usage by the assemblers on human K562-cells dataset.

Assemblers	CPU time (min)	Min_mem (G)	Max_mem (G)	Ave_mem (G)
TransComb	56	3.25	4.38	3.83
StringTie	23	3.88	4.56	4.02
Cufflinks	429	3.61	4.25	3.84
Bayesembler	537	3.51	9.64	4.69
